# Supplementary material for: The Association of HHV-6 and the TNF-α (-308G/A) Promotor with Major Depressive Disorder Patients and Healthy Controls in Thailand
Source: Viruses. 2023 Sep 8;15(9):1898. doi: 10.3390/v15091898 (PMC10535374; doi:10.3390/v15091898)

Supplement Table S1. Risk factor of MDD (HHV-6 negative 44 cases)

Factor	Group	N	MDD	Healthy	P-value	Odds ratio
Sex	Male	13	4(30.77%)	9(69.23%)	0.184	0.433 (0.123-1.524)
	Female	79	40(50.63%)	39(49.37%)		
Education level	No–High Vocational Certificate	19	13(68.42%)	6(31.58%)	0.044	2.935 (1429-8.224)
	>High Vocational Certificate	73	31(42.47%)	42(57.53%)		
Income/month ($)	300–1000	53	32(60.38%)	21(39.62%)	0.005	2.935 (1.004-8.583)
	No–299	39	12(30.77%)	27(69.23%)		
Familial relationship	Quarrel	10	10(100%)	0(0%)	0.000	2.412 (1.865-3.119)
	No Quarrel	82	34(41.46%)	48(58.54%)		
BMI	>25	31	19(61.29%)	12(38.71%)	0.065	2.280 (0.941-5.523)
	<25	61	25(40.98%)	36(59.02%)		
Congenital disease	Yes	36	30(83.33%)	6(16.67%)	0.000	15.000 (5.171-43.511)
	No	56	14(25.00%)	42(75.00%)		
Five main food groups	No	42	25(59.52%)	17(40.48%)	0.040	2.399 (0.881-4.642)
	Yes	50	19(38.00%)	31(62.00%)		
Exercise	No	46	26(56.52%)	20(43.48%)	0.095	2.022 (0.881-4.642)
	1–7 times/week	46	18(39.13%)	28(60.87%)		
Fresh fruit consumption (per week)	No–1 to 2 times	50	24(48.00%)	26(52.00%)	0.971	1.015 (0.447-2.308)
	3–7 times	42	20(47.62%)	22(52.38%)		
Vegetable consumption (per week)	No–1 to 2 times	27	13(48.15%)	14(51.85%)	0.968	1.018 (0.415-2.500)
	3–7 times	65	31(47.69%)	34(52.31%)		
High-fat food consumption (per week)	No–1 to 2 times	48	19(39.58%)	29(60.42%)	0.098	0.498 (0.217-1.143)
	3–7 times	44	25(56.82%)	19(43.18%)		
Fermented food consumption (per week)	3–7 times	9	7(77.78%)	2(22.22%)	0.058	4.351 (0.853-22.2081)
	No–1 to 2 times	83	37(44.58%)	46(55.42%)		
Cleaning of water for consumption	No	27	7(25.93%)	20(74.07%)	0.007	1.720 (1.203-2.459)
	Yes	65	37(56.92%)	28(43.08%)		
Tap water used for brushing teeth	Yes	79	42(53.16%)	37(46.84%)	0.012	6.243 (1.299-30.012)
	No	13	2(15.38%)	11(84.62%)		
Alcohol consumption	Yes	68	32(47.06%)	36(52.94%)	0.804	0.889 (0.350-2.255)
	No	24	12(50.00%)	12(50.00%)		
Secondhand smoke exposure	Yes	36	26(72.22%)	10(27.78%)	0.000	5.489 (2.187-13.773)
	No	56	18(32.14%)	38(67.86%)		

Supplement Table S2. Risk factor of MDD (HHV-6 positive 15 cases)

Factor	Group	Healthy	MDD	Healthy	P-value	Odds ratio
Sex	Male	5	2(40.00%)	3(60.00%)	0.635	0.500 (0.069-3.647)
	Female	21	12(57.14%)	9(42.86%)		
Education level	No–High Vocational Certificate	4	3(75.00%)	1(25.00%)	0.605	2.750 (0.248-30.512)
	>High Vocational Certificate	23	12(52.17%)	11(47.83%)		
Income/month ($)	300–1000	14	9(64.29%)	5(35.71%)	0.449	2.100 (0.448-9.836)
	No–299	13	6(46.15%)	7(53.85%)		
Familial relationship	Quarrel	5	5(100.00%)	0(0%)	0.047	2.222 (1.392-3.477)
	No Quarrel	22	10(45.45%)	12(54.55%)		
BMI	>25	10	7(70.00%)	3(30.00%)	0.424	2.625 (0.502-13.725)
	<25	17	8(47.06%)	9(52.94%)		
Congenital disease	Yes	17	12(70.59%)	5(29.41%)	0.057	5.600 (1.015-30.904)
	No	10	3(30.00%)	7(70.00%)		
Five main food groups	No	16	8(50.00%)	8(50.00%)	0.696	0.571 (0.119-2.751)
	Yes	11	7(63.64%)	4(36.36%)		
Exercise	No	13	11(84.62%)	2(15.38%)	0.006	13.750 (2.054-92.039)
	1–7 times/week	14	4(28.57%)	10(71.43%)		
Fresh fruit consumption (per week)	No–1 to 2 times	17	10(58.82%)	7(41.18%)	0.706	1.424 (0.297-6.877)
	3–7 times	10	5(50.00%)	5(50.00%)		
Vegetable consumption (per week)	No–1 to 2 times	13	7(53.85%)	6(46.15%)	1.000	0.875 (0.191-3.999)
	3–7 times	14	8(57.14%)	6(42.86%)		
High-fat food consumption (per week)	No–1 to 2 times	20	11(55.00%)	9(45.00%)	1.000	0.917 (0.161-5.207)
	3–7 times	7	4(57.14%)	3(42.86%)		
Fermented food consumption (per week)	3–7 times	3	3(100.00%)	0(0%)	0.231	2.000 (1.341-2.984)
	No–1 to 2 times	24	12(50.00%)	12(50.00%)		
Cleaning of water for consumption	No	9	6(66.67%)	3(33.33%)	0.683	2.000 (0.378-10.578)
	Yes	18	9(50.00%)	9(50.00%)		
Tap water used for brushing teeth	Yes	22	14(63.64%)	8(36.36%)	0.139	7.000 (0.663-73.929)
	No	5	1(20.00%)	4(80.00%)		
Alcohol consumption	Yes	17	9(52.94%)	8(47.06%)	1.000	0.750 (0.154-3.654)
	No	10	6(60.00%)	4(40.00%)		
Secondhand smoke exposure	Yes	10	7(70.00%)	3(30.00%)	0.424	2.625 (0.502-13.725)
	No	17	8(47.06%)	9(52.94%)		

Supplement Figure S1. Prevalence of HHV-6 infection and MDD patients in Asia.

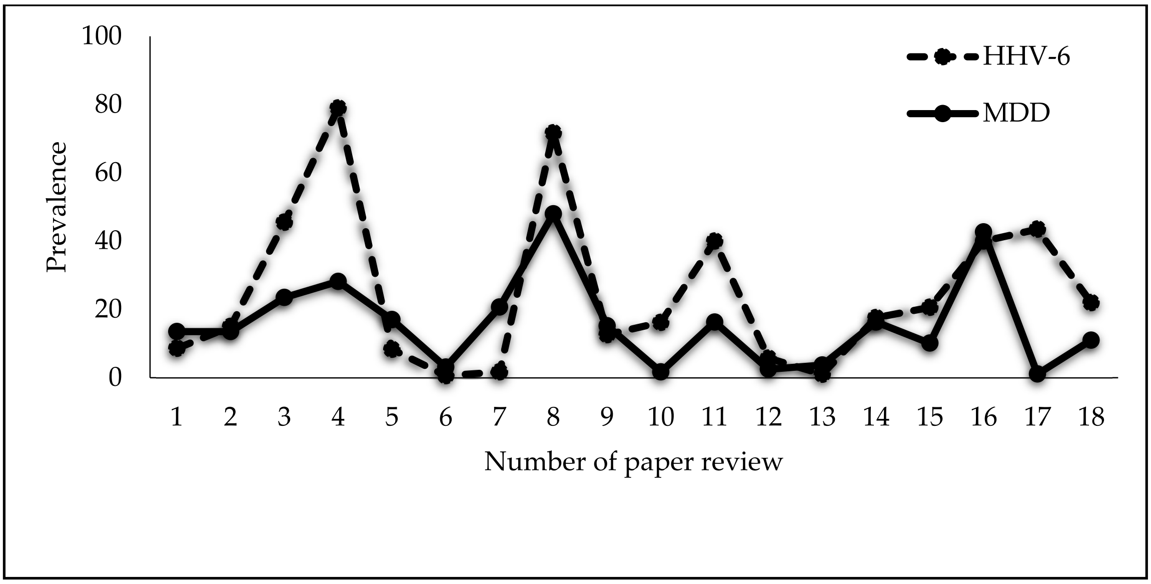

Supplement: Supplementary file 1 [file viruses-15-01898-s001.zip › viruses-2550996-supplementary.pdf]
